# Unexpected mixed-mode transmission and moderate genetic regulation of *Symbiodinium* communities in a brooding coral

**DOI:** 10.1038/s41437-018-0059-0

**Published:** 2018-02-17

**Authors:** Kate M. Quigley, Patricia A. Warner, Line K. Bay, Bette L. Willis

**Affiliations:** 1 ARC Centre of Excellence for Coral Reef Studies and College of Science and Engineering, James Cook University, Townsville, QLD Australia; 2 AIMS@JCU, Australian Institute of Marine Science and James Cook University, Townsville, QLD Australia; 3 Australian Institute of Marine Science, PMB3, Townsville, QLD Australia

## Abstract

Determining the extent to which *Symbiodinium* communities in corals are inherited versus environmentally acquired is fundamental to understanding coral resilience and to predicting coral responses to stressors like warming oceans that disrupt this critical endosymbiosis. We examined the fidelity with which *Symbiodinium* communities in the brooding coral *Seriatopora hystrix* are vertically transmitted and the extent to which communities are genetically regulated, by genotyping the symbiont communities within 60 larvae and their parents (9 maternal and 45 paternal colonies) using high-throughput sequencing of the ITS2 locus. Unexpectedly, *Symbiodinium* communities associated with brooded larvae were distinct from those within parent colonies, including the presence of types not detected in adults. Bayesian heritability (*h*^2^) analysis revealed that 33% of variability in larval *Symbiodinium* communities was genetically controlled. Results highlight flexibility in the establishment of larval symbiont communities and demonstrate that symbiont transmission is not exclusively vertical in brooding corals. Instead, we show that *Symbiodinium* transmission in *S. hystrix* involves a mixed-mode strategy, similar to many terrestrial invertebrate symbioses. Also, variation in the abundances of common *Symbiodinium* types among adult corals suggests that microhabitat differences influence the structure of *in hospite Symbiodinium* communities. Partial genetic regulation coupled with flexibility in the environmentally acquired component of *Symbiodinium* communities implies that corals with vertical transmission, like *S. hystrix*, may be more resilient to environmental change than previously thought.

## Introduction

Symbiosis is fundamental to life on Earth, underpinning the existence of numerous prokaryotic and eukaryotic species and shaping the physiology and health of many organisms (Moya et al. 2008; Gilbert et al. 2012; Lewis et al. 2015). Microbial symbionts also enable hosts to expand their niche breadth to survive in environments otherwise unsuited to their physiology (Goffredi et al. 2007). For example, symbiosis with photosynthetic dinoflagellates of the genus *Symbiodinium* has allowed corals to thrive in oligotrophic tropical seas through the utilization of symbiont photosynthates. Similar nutritional facilitation has been described for sap-sucking insects that rely on microbial partners to supplement their diets (Baumann 2005). Compared to these well-characterized systems, coral endosymbioses are poorly described at the *Symbiodinium*-type level during early ontogeny.

Nutritional symbioses can drive diversification of host and symbiont lineages (Douglas 1989; Brucker and Bordenstein 2012; Oliver et al. 2014), with eukaryotic symbionts like *Symbiodinium* that have gone through multiple cycles of diversification and expansion (Thornhill et al. 2014). This standing genetic variation provides new material upon which selection may operate (Moran et al. 2008; Russell et al. 2012), facilitating coevolution between hosts and symbionts or among symbionts (Moran and Dunbar 2006; Moran et al. 2008; Moya et al. 2008). Understanding the fidelity (here defined as the exactness of transfer of symbionts from parent to offspring) of *Symbiodinium* community inheritance is key to determining the degree to which endosymbiotic *Symbiodinium* communities have coevolved with their coral hosts and is central to coral nutrition and health. Despite this, little is known about genetic regulation underpinning this symbiosis.

Symbionts may be acquired from the environment (horizontal transmission) or passed maternally into eggs or larvae (vertical transmission), with the latter thought to be the most prevalent mode of transmission in brooding scleractinian corals (Baird et al. 2009). Maternally derived symbionts may involve the transmission of one or multiple symbionts (superinfections), at least in well-studied insect vertically transmitting symbioses. Transmission of bacterial symbionts in insects may be exclusively vertical or may occur initially as vertical transfer followed later by horizontal transmission (Fujishima and Fujita 1985; Sandström et al. 2001; Kaltz et al. 2003; Scheuring and Yu 2012; Andersen et al. 2013; Oliver et al. 2014). Although similar mixed-mode transmission has been hypothesized for corals (Byler et al. 2013), the absence of experimental data means that it is not yet clear if transmission is exclusively vertical in brooding corals or if mixed-mode transmission also occurs in this group. Given recent evidence of differences in the diversity of symbiont communities transmitted from parents to offspring in two broadcast-spawning species (Padilla-Gamiño et al. 2012; Quigley et al. 2017a, 2017b), *Symbiodinium* transmission dynamics in corals may be as complex as those observed in the Arthropoda.

In general, symbiont-host specificity is theorized to be much greater when symbionts are transmitted vertically compared to horizontally (Douglas 1998; Baker 2003). In corals, hosts may form strict associations with only one *Symbiodinium* type (and vice versa) or associate with multiple partners, and superinfections of multiple *Symbiodinium* types and subtypes of varying abundances are common among species (Little et al. 2004; Abrego et al. 2009a, 2009b; Fabina et al. 2012; Byler et al. 2013; Poland and Coffroth 2017). Although maternal transfer of *Symbiodinium* and bacteria is less well-characterized in corals than in terrestrial invertebrates (Apprill et al. 2009; Padilla-Gamiño et al. 2012; Sharp et al. 2012; Byler et al. 2013, 2017a, 2017b), the presence of superinfections raises the possibility that *Symbiodinium* dynamics are similar to the mixed-mode transmission dynamics characteristic of superinfections described in terrestrial invertebrates like aphids and sharpshooter cicada (Moran et al. 2008; Oliver et al. 2014). However, unlike studies of insect symbiont specificity, no studies have used high-throughput sequencing to examine maternally transmitted *Symbiodinium* communities in brooding corals or the diversity of low abundance *Symbiodinium* types in detail. Similarly, the genetic component of parental contributions to the maturation of coral-*Symbiodinium* symbioses remains unquantified.

It is clear that *Symbiodinium* types vary in their impact on holobiont physiology because of variation in their stress tolerance and ability to produce and transfer photosynthates to the coral host under differing light, temperature and nutrient regimes (Little et al. 2004; Berkelmans and van Oppen 2006; Reynolds et al. 2008; Cantin et al. 2009; Hume et al. 2015; LaJeunesse et al. 2015). Moreover, environmental variation and stress may bring about shifts in the dominance of *Symbiodinium* types, in some cases benefiting the host under the altered conditions (Jones et al. 2008; Cunning et al. 2015). The extent of a coral’s flexibility to acquire resilient types or shuffle symbionts may be genetically regulated, for example by heritable host immune responses, similar to those that shape symbiont diversity in *Drosophila* (Mateos et al. 2006). Complete inheritance results in complete fidelity of symbiont transmission, and, hence, little scope for flexibility in coral-*Symbiodinium* symbioses. However, the extent of such potential regulation of symbiont transmission and its underlying basis are unknown for corals.

It is increasingly revealed that the genetic architecture behind traits and pathologies can be complex (Cho 2015). For example, both the diversity and abundance of microbial symbionts in the human gut are complex traits under partial genetic control (Zoetendal et al. 2001; Ley et al. 2006; Benson et al. 2010; Turnbaugh et al. 2010; Campbell et al. 2012; Liu et al. 2015). Narrow-sense heritability (*h*^2^) is the parameter typically used to describe the degree to which variability in a trait is explained by genetic factors. Assuming that the *Symbiodinium* community associated with a coral can be represented as a complex trait, then an *h*^2^ value of 1 would imply that variability of the community is mostly due to host genetics. Conversely, an *h*^2^ value estimated at 0 would imply no genetic basis for variability in the community, thus the community would not be under selection and could not evolve (no evolvability; Lynch and Walsh 1998). Although an *h*^2^ estimate close to 1 does not necessarily guarantee absolute genetic determination as a result of gene segregation (Visscher et al. 2008), a large heritability estimate of the *Symbiodinium* community would imply that changes in host genotypes are required for shifts in symbiont communities. Conversely, changes in the environmental availability of *Symbiodinium* or in environmental conditions would have limited influence on *in hospite* communities. Understanding the relative contributions that host genetics versus environmental conditions make to the composition of *Symbiodinium* communities through estimations of *h*^2^ will improve the accuracy with which the potential, direction and speed of changes in *Symbiodinium* communities can be predicted.

To examine *Symbiodinium* community transfer between adults and their offspring in a brooding coral and quantify the narrow-sense heritability (*h*^2^) of this trait, we quantified the *in hospite Symbiodinium* communities of individual planula larvae and their parents across a spectrum of relatedness using high-throughput sequencing. Relatedness was based on a population genetic parentage analysis that assigned the likely paternal identity of each larva. In light of results on heritability and fidelity of symbiont transfer, we discuss the potential of larvae from brooding corals like *S. hystrix* to acclimate to novel environments.

## Materials and methods

### Study species and sampling design

The common, hermaphroditic coral *Seriatopora hystrix* broods sexually produced larvae following internal fertilization of eggs by sperm from surrounding colonies (Ayre and Resing 1986; Warner et al. 2016). DNA extracts of planula larvae for the present study were selected from samples that were collected in an earlier study to assess sperm dispersal distances and larval parentage of a cryptic species within the *S. hystrix* species complex, specified as *S. hystrix* (ShA) (Warner et al. 2015, 2016). In Warner et al.’s study, colonies were tagged and sampled for molecular analyses within a 16 m × 16 m sampling area, with additional colonies sampled from two adjacent transects (totaling 16 m × 40 m area) in the Lizard Island lagoon (S14°41.248, E145°26.606; Warner et al. 2015, 2016). Microsatellite genotypes and paternity assigned to individual larvae in this earlier study (Warner et al. 2016) enabled us to examine the effect of both maternal and paternal identity on larval *Symbiodinium* communities across a full pedigree of larval relatedness. Hence, our study included full-sib and half-sib larvae, and four individuals produced by selfing (further details in supporting information and Table S1).

### *Symbiodinium* community genotyping

*Symbiodinium* communities of adults and larvae were quantified with amplicon sequencing of the ITS2 locus using the same DNA extractions that had been used to assign microsatellite genotypes and paternity in Warner et al. (2016). Maternal and paternal identities of each of the 60 larvae were estimated in (Warner et al. 2016). Specifically, 9 maternal and 45 assigned paternal colonies (which included the nine maternal colonies), plus all larvae whose paternity was designated with a confidence level of very high (95% posterior paternity probability + 95% confidence assignment), high (95 + 80%), or medium (95% only) by Warner et al. (2016) (*n* = 60 larvae) were sequenced with the primers ITS2alg-F and ITS2alg-R (Pochon et al. 2001) using paired-end Illumina Miseq technology. Library preparation and sequencing were performed at the University of Texas at Austin’s Genomics Sequencing and Analysis Facility (USA) using their standard protocols, including Bioanalyzer (Agilent)-based DNA standardization and pooled triplicate PCR before library preparation.

Raw reads (total = 6,875,177) were analyzed using the USEARCH and UPARSE pipelines (v.7; Edgar 2013), as outlined in Quigley et al. (2016) (further details in supporting information). Because there is currently no single copy marker for *Symbiodinium* genotyping (Pochon et al. 2012), the ITS2 marker was selected for the broadest comparisons to the vast literature that has used this marker to describe *Symbiodinium* diversity, including some using next generation sequencing (e.g., Arif et al. 2014; Green et al. 2014; Thomas et al. 2014; Quigley et al. 2016, 2017a, 2017b; Ziegler et al. 2017a). Additional steps were taken to assess the presence and impact of intragenomic variants (further explained below). Briefly, reads were filtered, clustered into OTUs at 97% similarity, annotated with NCBI nt database and *Symbiodinium*-specific searches (further details in Table S2). Using these methods, the majority of the OTUs were re-assigned to a clade/type level, leaving only 0.03% of cleaned reads (1459 reads, 78 OTUs) that could not be classified, and which may represent new *Symbiodinium* types (Table S3, Fig. S1, supporting information).

To account for variable read-depth across all samples, sample reads were normalized using “DESeq2” and “Phyloseq” implemented in R (R Core Team 2012; McMurdie and Holmes 2013; Love et al. 2014a, 2014b). Nonmetric multidimensional scaling (NMDS) was performed and plotted using the normalized counts matrix using “Phyloseq”, “vegan”, and “ggplot” (Wickham 2009; Schloerke et al. 2014). A permutational multivariate analysis of variance and permutation test for homogeneity of multivariate dispersions were used to determine whether significant differences in *Symbiodinium* community structure exist between broods using the “adonis” and “betadisper” functions in “vegan.” Genetic distances between OTUs were calculated in “Ape” (Paradis et al. 2004). Statistical testing of variation in OTU abundance was performed on raw reads in “DESeq2”, which incorporates variance normalization of OTU abundance, and interpreted using the Bejamini–Hochberg correction for multiple-inferences of *p*-adjusted alpha at 0.05. DESeq2 calculates *p*-values based on independent filtering criteria and Benjamini–Hochberg multiple testing adjustments to determine whether *p*-values are significant. “DESeq2” outputs are expressed as the mean of normalized counts of treatment groups (baseMeans) and multiplicative (log_2_ fold) terms between or among treatments (Love et al. 2014a, 2014b). Network analysis on planula larvae was performed using the “igraph” package (Csardi and Nepusz 2006) and custom scripts from (Cunning et al. 2017).

### Estimating the diversity and heritability of *Symbiodinium* communities

To describe the *Symbiodinium* community in coral samples, we used a diversity measure (^*q*^*D*^*Z*^_*ij*_(*p*)) that incorporates OTU richness, evenness, and sequence similarity (Leinster and Cobbold 2012). This metric therefore combines a variance-normalized matrix of OTU abundances and diversity, rarity, and a matrix of pairwise similarities between sequences (sequence similarity). Sequence similarity was calculated using pairwise percent similarities between OTU sequences using the “Ape” package with a “raw” model of molecular evolution. Through this diversity metric (further described in Quigley et al. 2017a), the *Symbiodinium* community is presented as a continuous quantitative host trait. Heritability of *Symbiodinium* diversity associated with the 60 larvae was calculated using the package “MCMCglmm” (Hadfield 2010) utilizing the diversity metrics described above, where the coefficient of relatedness between individuals was set as a random effect. The coefficient of relatedness is the degree to which individuals share genetic material. For example, full siblings share 50% of their genetic material, whereas clones would share 100%. Maternal environmental effects and paternal effects were assessed and were not significant. Deviance Information Criterion was used to test if adding a maternal random effect had a statistically significant effect on heritability estimates. Models were run with 1.5 × 10^6^ iterations, a thinning of 50, and burn-in of 10% of the total iterations. A non-informative flat prior specification was used following an inverse gamma distribution (see Wilson et al. 2010 for further analysis details and scripts). Assumptions of chain mixing, normality of posterior distributions, and autocorrelation were met. The posterior heritability was calculated by dividing the model variance attributed to relatedness by the sum of additive and residual variance.

### Multiple ITS2 copies and intragenomic variation

Intragenomic variation within and between *Symbiodinium* types makes classifying type-level diversity in *Symbiodinium* based on sequence data difficult (Thornhill et al. 2007; Sampayo et al. 2009; Arif et al. 2014; Quigley et al. 2014). We addressed intragenomic variation by clustering across samples at 97% similarity and provide two additional analyses to test for their presence and potential impact on the heritability estimate; and both confirm the robust nature of our conclusions in regards to this issue (Supporting Methods and Results).

### Colony size and spatial distribution of adult *S. hystrix* (ShA) colonies

To determine if *Symbiodinium* communities varied with colony size (as a proxy for colony age), adult colonies were divided into five size classes based on their mean diameter (Warner et al. 2016): <8 cm (*n* = 1 colony), 8 – <14 cm (*n* = 19), 14 – <20 cm (*n* = 13), 20 – <26 cm (*n* = 11), and 26–32 cm (*n* = 1). Differential abundance testing of *Symbiodinium* OTUs among size classes was performed as for larval communities.

Sitepainter (Gonzalez et al. 2012) and Inkscape (Bah 2009) were used to visualize spatial patterns in the distribution of *Symbiodinium* OTUs associated with the 45 adult colonies of *S. hystrix* (ShA) that were genotyped across the 16 m × 40 m sampling area. Gradient-boosted models and linear models were run in the package “gbm” (Ridgeway 2006) to examine spatial distributions of the ten most abundant OTUs. These types of Boosted Regression Trees uses machine learning and predictive modeling to construct models, thereby making them particularly suited to spatially patchy data of *S. hystrix* colonies in our sampling area (Leathwick et al. 2008). Linear models were checked for assumptions of linearity, normality, and homogeneity of variance. Square-root transformations were used to correct for issues of normality or heterogeneity. Latitude and longitude coordinates were centered before fitting models. The package “Spatstat” (Baddeley and Turner 2005) was used to visualize spatial variability in abundances of the three most significantly heterogeneous OTUs across the sampling area (OTUs: 1, 3, and 6). Spearman’s rho rank correlation coefficients were calculated to test for competitive exclusion amongst the three OTUs that varied significantly across the sampling area. Pairwise *p*-values were generated for all OTU comparisons using the base “stats” package in R.

## Results

### *Symbiodinium* communities differ between parents and brooded larvae

**Fig. 1:**
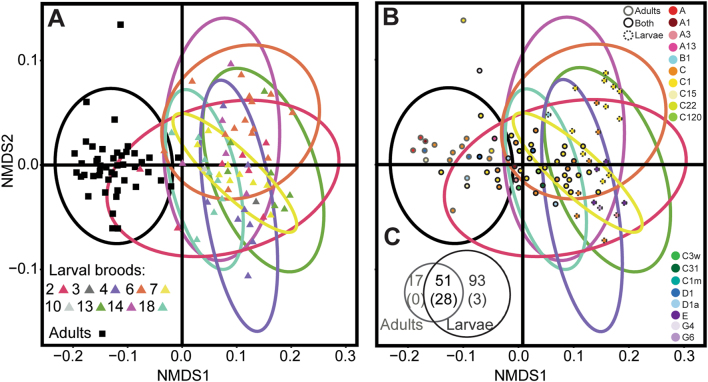
Nonmetric multidimensional scaling (NMDS) Split Biplot, based on a Bray–Curtis distance matrix of variance-normalized OTU abundances and sequence similarity between OTUs (pairwise percent identities), illustrating differences between *Symbiodinium* communities associated with adult colonies and larvae of the brooding coral *Seriatopora hystrix* (ShA) based on parentage analysis. Ellipses encircling symbols of the corresponding color represent 95% probability regions for adults (black) and larval broods (colored), where each brood represents all larvae sharing the same dam (color-coded). Samples and OTUs have been separated to increase Biplot clarity whilst maintaining ellipse positions to facilitate comparisons of ordination space between samples and OTU positions. **a** Each point represents the *Symbiodinium* community associated with a unique coral adult or larval sample. **b** Each point represents an OTU colored by type level. Only 83 of the 161 OTUs are shown, representing those OTUs with the greatest taxonomic confidence (see Table S7 for full names and selection criteria). Outlining around each point represents the origin of the OTU, i.e., those found uniquely in adult (gray outline) or larval (broken gray outline) samples, or retrieved from both (black outline). Samples presented in **a** and OTUs presented in **b** share the same ordination space but were separated for clarity. **c** Venn diagram, illustrating the number of *Symbiodinium* OTU’s that were unique to larvae (dark gray text) versus adults (light gray text). The number of OTUs that were significant after p-adjustments are in parentheses. Ellipses corresponding to dams 3 and 10 are not represented, as only one larva per dam was collected and sequenced

**Fig. 2:**
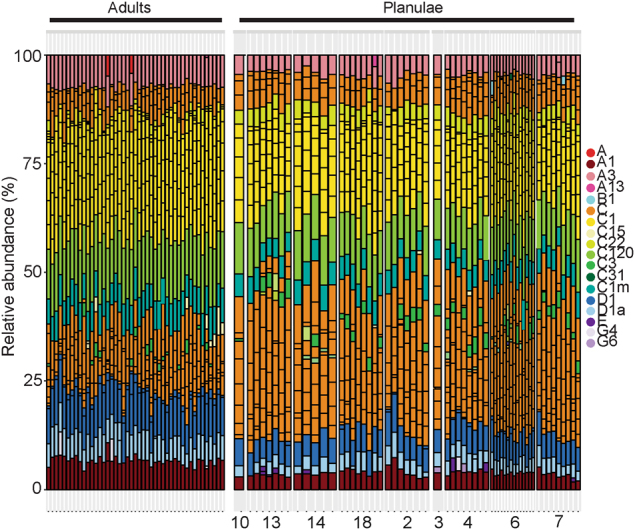
Barplots of variance-normalized abundances of *Symbiodinium* diversity associated with **a** adults and **b** planula larvae of *Seriatopora hystrix*. Colors represent different *Symbiodinium* types. Numbers below planulae barplots represent the different broods

*Symbiodinium* communities differed between adults and their larvae in the brooding coral *Seriatopora hystrix* (ShA) (Fig. 1a, b). Overall, the composition of *Symbiodinium* communities was significantly different between adults and larvae, with higher similarity among adult corals, but more variable among larvae (Permutation test for homogeneity of multivariate dispersions, df = 1, *p* *= *0.001). On average, adults contained 29.9 ± 0.6 (SE) OTUs and larvae had 22 ± 0.4 OTUs (Fig. 2). However, the number of unique OTUs recovered was more than five times greater from larvae than from adults (93 vs. 17 OTUs, respectively; Fig. 1c). Of the 17 unique adult OTUs, ten belonged to clade C (C1, C15, and other variants), three from clade A (A1 and variants), three from D (including D1 and D1a), and one was a putative C type (Fig. 1b). Unique to larvae were 17 OTUs from clade C (likely C1 variants), four from clade E, one from each of A3, B1, and G6, and 69 that were of putative *Symbiodinium*-type level identity (Fig. 1b). Of the 93 larval-specific OTUs, only the abundance of C1_OTU136 (type followed by OTU designation) and two putative clade D OTUs (OTU148 and OTU149) were significantly different from zero with the Bejamini–Hochberg correction (Fig. 1b). Although raw read counts were low, C1_OTU136 was present in larvae from every dam but dam 3.

**Fig. 3:**
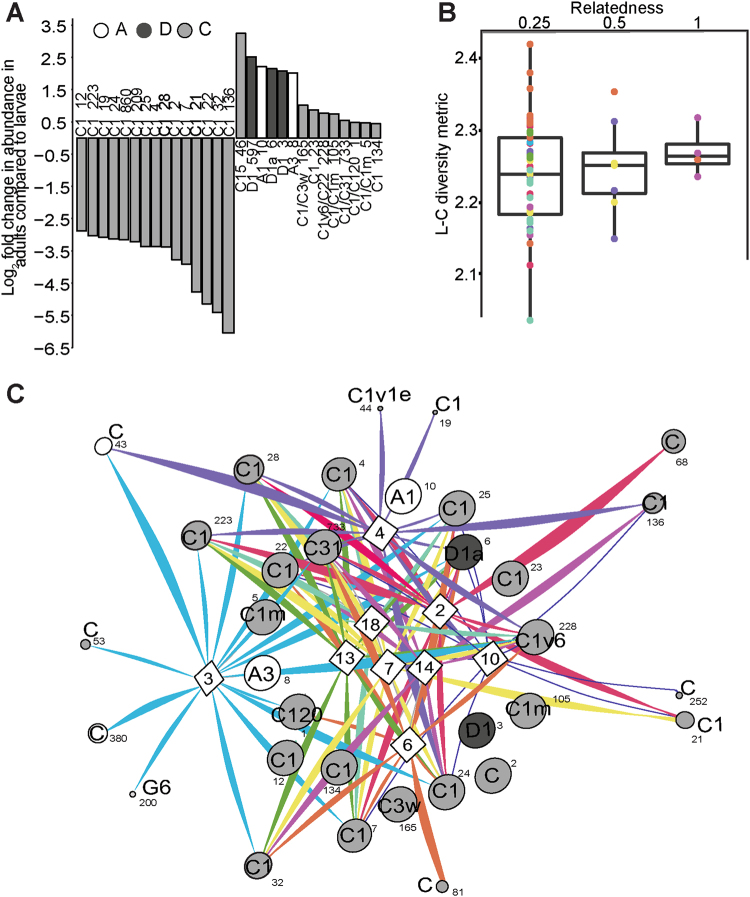
**a** Log_2_ fold change in abundances of *Symbiodinium* OTU’s that differed significantly between communities associated with adults versus larvae of *Seriatopora hystrix* (ShA). Gray-scale in the bar plot identify *Symbiodinium* clades. A positive change indicates the OTU is more abundant in adults. **b** Boxplots showing medians, quartiles, and minimum/maximum values of *Symbiodinium* community diversity (Leinster and Cobbold metric) in relation to individual larval relatedness. On the *x*-axis, 0.25 denotes half sibs, 0.5 full sibs, and 1.0 denotes larvae produced from selfing. Each larva is colored by its respective dam. **c** Network analysis of planula larvae showing OTUs present in 50% or more of larvae per brood. Edges have been removed for OTUs found in 100% of larvae from each brood (OTUs:1, 2, 3, 5, 8, 10, 12, 23, 105, 134, and 165). White diamonds correspond to maternal broods, where each brood sharing the same dam is color-coded. Small numbers next to each node indicate the OTU number for that *Symbiodinium* type. Line thickness denotes relative abundance of the *Symbiodinium* type per brood

Fifty-one OTUs were shared by adult colonies and planula larvae (43 of known *Symbiodinium*, 8 of putative *Symbiodinium* taxonomy, Fig. 3a), and the abundance of 28 of these OTUs differed significantly between the two groups at the adjusted p-level, including both dominant (i.e., C1/C120_OTU1) and background OTUs (C15_OTU46) (Table S4). Of these 28 OTUs, 23 were from clade C (including C1, C3w, C120 among others), three from clade D (D1, D1a), and two from clade A (A1, A3). Adult *Symbiodinium* communities were characterized by up to 4–5.7 (2–2.5 log_2_ fold) times more D-types (D1_OTU3, D1_OTU597, D1a_OTU6), and A-types (A1_OTU10 and A3_OTU8) compared to larvae (Bejamini–Hochberg corrections, Table S4). Nine of the 23 C-types had up to 9.2 times (3.2 log_2_ fold) significantly higher abundances in adults (including multiple C1 types, C120/C120a_OTU1, C1m_OTU5/105, C1v6/C22_OTU228, C15_OTU46, C31_OTU733, and C3W_OTU165) and the remaining C1 types had between 0.02–0.13 (−5.4 to −2.9 log_2_ fold) (times significantly lower abundances in adults (Table S4). D1, D1a, A1, and A3 were found at high relative abundances in adult and larval communities (Fig. 2, Table S4). C1/C120 OTUs were detected at the greatest mean relative abundances. In contrast, some of the C1/C15 OTUs were detected at mean relative abundances at the background level (<0.16, Table S4).

### Larval *Symbiodinium* communities vary among broods

Planula larvae that shared the same maternal parent generally grouped together when *Symbiodinium* OTU richness, abundance, and DNA distance between OTUs were incorporated into analyses. Maternal broods differed significantly in their *Symbiodinium* community structure (permutational analysis of variance by dam; *R*^2^ = 0.57, df = 44, *p* = 0.001) although 95% probability regions overlapped (Fig. 1a, b). Thirty-one OTUs (including multiple C1 variants, D1, D1a, A1, and A3) were found in >50% of larvae per brood and were generally present across all broods (Fig. 3c) and drive the structure among larval broods.

Eleven OTUs dominated larval communities and were found in all larvae from all broods, including C120, C1m, and D1 (see full OTU list in Fig. 3c). Other OTUs contributed to the unique structure of each brood and included OTUs that were highly prevalent within broods of highly related larvae (e.g. brood 3 and 4: G6 and C1v1e, respectively). At least 8 of these key OTUs were not shared between broods (i.e., OTUs 81, 252, 68), and highlight the distinctiveness of larval samples across broods and contributed to full-sib phenotypic correlations compared to those of half sibs. Alternatively, OTUs that are prevalent across all broods (e.g., C120, D1, A3, and A1) contributed to structuring between larval broods (overlap between OTUs shared between broods). Furthermore, OTUs also varied in their relative abundance across larval broods, with particular OTUs being numerically dominant in specific broods compared to others, such as C_68 in brood 2 and C_81 in brood 6 (Fig. 3c).

However, differences in the abundance of OTUs amongst larval broods were detected for symbiont types A1, A3, C1, D1, and D1a, amongst others. Briefly, larvae from dam 2 displayed higher abundances of A1 and A3. Larvae from dams 3, 7 and 10 had significantly less of C1_OTU2, whilst broods from dams 4, 6, 13, 14, and 18 had significantly different abundances of many C-types, including C120/C120a, C1, C1v1e, C1m, and C31. The abundances of D1_OTU3 and D1a_OTU6 also varied significantly among larval broods, particularly among those from dams 2, 4, and 18 vs. dam 13 (for a full description see supporting information and Table S5).

### Heritability

Leinster and Cobbold estimates of *Symbiodinium* community diversity varied across the 60 larvae. Notably, more closely related larvae had more similar *Symbiodinium* communities (Fig. 3b). The posterior mean heritability of the *Symbiodinium* community in *S. hystrix* (ShA) larvae was 0.43 ± 0.21 SD, with a posterior mode of 0.33 (95% Bayesian credibility interval (BCI) 0.1–0.8; Fig. S2, supporting information). The effect of larvae sharing the same maternal environment was not significant (no model improvement; Deviance Information Criterion <2 units with the addition of maternal identity as a random effect) but decreased the posterior mean and mode of heritability slightly (mean = 0.37 ± 0.21 SD and mode = 0.19; BCI: 0.1–0.8).

### Patterns in adult *Symbiodinium* communities of varying spatial distribution and colony size

**Fig. 4:**
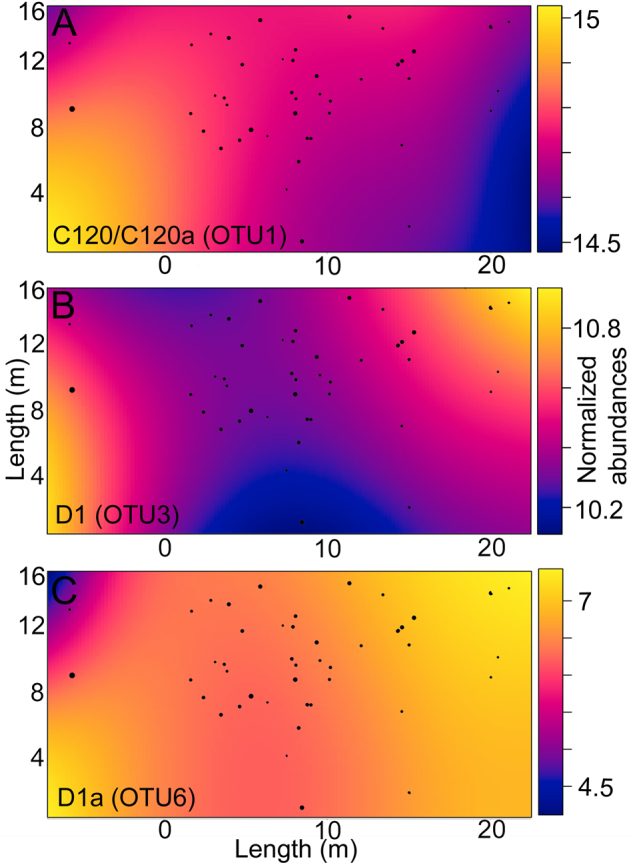
Spatial patterns in the normalized abundance of three *Symbiodinium* OTU’s associated with adult colonies of *Seriatopora hystrix* (ShA) that differed significantly in their abundances across a portion of the 16 m × 40 m sampling area at Lizard Island. Positions of the 45 genotyped adult colonies are denoted by black circles. **a** C120/C120a, **b** D1, and **c** D1a. Colors represent changes in the normalized abundance of each OTU across sampling site coordinates, with yellow representing the highest abundance and blue the lowest. Sizes of the black circles represent size classes of coral colonies in cm drawn to scale of the sampling area (smallest circles are 10 cm and the largest are 30 cm)

The distributions of three of the ten most abundant OTUs in adult corals varied significantly across the sampling area (*p* > 0.05; Fig. 4), although not in a consistent manner with distance either along or down the sampling area. For example, although abundances of *Symbiodinium* C120/C120a were greatest in colonies that were closest to the lower left of the sampling area (corresponding to 55,737–29,885 non-normalized reads, the most abundant *Symbiodinium* OTU in *S. hystrix*; gradient-boosted model (GBM): *p* = 0.019), consistent with a gradual increase in distance down the reef slope, this pattern was not consistent along the reef slope (GBM: *p* = 0.00841). The abundance of D1 was significantly higher in the top-right and lower left side of the sampling area than in other aspects (*x* and *y* interaction, corresponding to 7802–800 non-normalized reads; GBM: *p* = 0.0393). D1a was least abundant in the top left and inner portion of the sampling area (corresponding to 876-0 non-normalized reads; GBM: *p* = 0.0405). Finally, although the variance normalized abundances of all three OTUs were significantly positively correlated overall (Spearman’s rank correlation *ρ*: 0.42–0.77, all *p* < 0.004), extremely low abundances of C120/C120a at *x*-coordinates >15 contrast markedly with high abundances of the two D-types in the same region (Fig. 3). Of the 68 *Symbiodinium* OTUs found in adults, the abundance of only C1_OTU4 differed significant among the five coral size classes, with colonies from the 8–14 cm size class hosting 1.7 times lower C1 abundances compared to corals in the 14–20 cm size class (*p* > 0.05; Table S6).

## Discussion

### Mixed-mode transmission structures larval *Symbiodinium* communities in a brooding coral

The availability of a full larval pedigree for *Seriatopora hystrix* (ShA) (Warner et al. 2016) provided a unique opportunity to evaluate the relative contributions of heritability (i.e., the degree to which variability in a trait is explained by genetic factors) versus maternal environmental effects (the effect of larvae sharing a common maternal environment) to the composition of larval *Symbiodinium* communities in a brooding coral. Here we show that *Symbiodinium* communities associated with larvae of *S. hystrix* (ShA) differ from those associated with their parents, providing experimental evidence that at least a portion of the *Symbiodinium* community is horizontally transmitted in a brooding coral. These results further demonstrate that coral-*Symbiodinium* symbioses align with well-characterized models of invertebrate symbioses. Results from previous studies suggesting exclusively vertical transmission in brooding corals may have been due to the lower detection capability of earlier methods and will no doubt change with the continued application of NGS technology to more brooding coral species. Overall, *Symbiodinium* communities were found to be moderately heritable, with only 33% of variability in larval symbiont communities under genetic regulation. Model selection also showed that, for larvae released from the same colony, sharing the same maternal environment did not significantly explain variability in *Symbiodinium* communities found among larvae from the same brood. This result, combined with the moderate heritability estimate, indicates that similarities in *Symbiodinium* communities among larvae of the same maternal brood were due to gene(s) inherited by these larvae.

Heritability estimates reveal important information about the evolvability of a trait, such as the capacity of brooding corals to vary their symbiont communities in response to changing environmental conditions. If levels of heritability and genetic variance are low, then responses to natural or artificial selection (evolvability) would be limited (Visscher et al. 2008). Conversely, high heritability and high genetic variance of a trait would enable greater responses to selection pressures. On the other hand, highly heritable symbiont communities with low genotypic variation could be problematic for vertically transmitting coral populations if adult communities are thermally sensitive (Matz et al. 2017). We found moderate heritability of *Symbiodinium* communities in *S. hystrix* (ShA). Much greater heritability of the *Symbiodinium* community was expected in this vertically transmitting coral, and in comparison with what is known of other important reproductive and fitness traits. For example, fertilization success, larval heat tolerance, protein content, settlement success, settlement substrate preferences, and juvenile growth and survivorship are all heritable traits (Meyer et al. 2009; Kenkel et al. 2011, 2015; Baums et al. 2013; Dixon et al. 2015). Although the distribution of posteriors was skewed towards values greater than that of our heritability estimate, it is unlikely that heritability (i.e., genetic regulation) for this trait will resolve to be much greater with increased sampling effort (~0.5–0.6, Fig. S2). The moderate levels of genetic regulation (i.e., heritability) found here suggest that *S. hystrix* (ShA) has some capacity to respond to changing environmental conditions. Thus, intervention efforts to facilitate such phenotypic change may be possible (Visscher et al. 2008). Given that assisted evolution efforts involving heat-selected *Symbiodinium* types show promise in horizontally transmitting corals (Levin et al. 2016), it may be that vertically transmitting, brooding species with moderate fidelity like *S. hystrix* (ShA) could also be candidates for assisted *Symbiodinium* uptake.

### Combined maternal and environmental uptake produces locally adapted but flexible *Symbiodinium* communities

Detection of 93 larval-specific OTUs in this study demonstrates that brooding corals like *S. hystrix* (ShA) have a mixed-mode transmission strategy, in which dominant symbionts are transmitted vertically but additional background strains are acquired from environmental sources. Although adult diversity may have been under-sampled by only sequencing one branch of each parental colony, unique larval OTUs were not detected in any of the 45 adult colonies that were genotyped. Environmental uptake of novel *Symbiodinium* by larvae of this species is further supported by the appreciable amount of variation in the composition of larval *Symbiodinium* communities that was not under genetic control, according to our heritability model. These results validate the hypothesis of potential mixed-mode transmission initially raised by Byler et al. (2013) although they did not find differences in diversity between *S. pistillata* adults and larvae (Byler et al. 2013). Finally, although many of the OTUs unique to larvae or significantly differentially abundant between life stages were at background abundances, rare *Symbiodinium* have important functional roles in regulating host fitness (Quigley et al. 2017b; Ziegler et al. 2017b).

Evidence of mixed-mode transmission in *S. hystrix* (ShA) contradicts previous assumptions that maternally transmitted symbiont communities are transferred to offspring with high fidelity in corals (Douglas 1998; Baker 2003; Fabina et al. 2012). Our findings are consistent with transmission patterns documented in other symbiotic systems, such as wild *Drosophilia hydei* populations (Oliver et al. 2014), *Acromyrmex* ants (Scheuring and Yu 2012; Andersen et al. 2013), and paramecium (Fujishima and Fujita 1985; Kaltz et al. 2003), and aligns symbiotic transmission ecology in corals with terrestrial invertebrate symbioses. In addition, the novel diversity found in *S. hystrix* (ShA) larvae mirrors increased diversity of *Symbiodinium* communities detected in eggs of *Montipora capitata* and *M. digitata* compared to adults (Padilla-Gamiño et al. 2012; Quigley et al. 2017a). Similarly, novel, bacterial community diversity was detected in the larvae of the brooding coral *Porites astreoides*, and various bacterial communities associated with larvae of sponge species with supposed vertical transmission (Schmitt et al. 2008; Sharp et al. 2012).

Mounting evidence for mixed-mode transmission across phyla suggests that it may be evolutionarily advantageous to compromise between completely vertically and horizontally acquired symbiont communities, as both strategies provide distinct advantages and disadvantages (Baird et al. 2009; Byler et al. 2013). In *S. hystrix* (ShA), vertical transmission of *Symbiodinium* that are locally adapted to the parental environment is likely to provide benefits for a species that is able to self-fertilize (Sherman 2008; Warner et al. 2016) and has highly localized larval dispersal (e.g., Underwood et al. 2007; van Oppen et al. 2008; Noreen et al. 2009). However, a locally adapted community might become a liability if environmental conditions change or if larval dispersal distances are long. Negative effects include deregulation or disruption of symbiont abundances, which may have harmful physiological effects on the host (Xie et al. 2010; Oliver et al. 2014; Cunning et al. 2015). Thus, a mixed-mode strategy that results in superinfections of multiple symbionts can be beneficial (e.g., parasitoid protection in aphid hosts, Sandström et al. 2001; Oliver et al. 2014) and may provide more flexibility for adjusting to variable environmental conditions. Similarly, a mixed mating strategy of selfing and outcrossing in *S. hystrix* (ShA), combined with a functional, established symbiosis upon release, may facilitate both local and long-distance dispersal (Warner et al. 2016). Our findings confirm that diversity and flexibility of *Symbiodinium* transmission in a brooding coral are greater than previously thought, highlighting the potential for evolvability that may confer greater resilience over time than coral species with strict vertical transmission.

Additional to environmental uptake of *Symbiodinium* during early ontogeny and processes like competitive exclusion may contribute to differences between larval and adult communities in *S. hystrix* (ShA). Theory suggests that competition among symbionts may preclude transmission of an exact replica of the parental symbiont community because conditions promoting growth for some symbionts may differ between life stages (Moran et al. 2008). The novel symbiont diversity found in *S. hystrix* (ShA) larvae may provide benefits similar to those observed in insect symbioses, for example to provide larvae with the flexibility to host optimal symbiont types for the changing conditions through ontogeny (Abrego et al. 2009b; Byler et al. 2013). For example, *Symbiodinium* C1_OTU136, which was uniquely identified in larvae, may represent an adaptive advantage for this early life stage. Clade C-types are taxonomically and physiologically diverse (LaJeunesse 2005; Thornhill et al. 2014), and exhibit a range of tolerances for light and temperature, which are also reflected in their *in hospite* distributions across individual adult colonies and species (Sampayo et al. 2008). Larval settlement and early juvenile survival are generally highest in cryptic, low-light areas that offer protection from predation (Maida et al. 1994; Suzuki et al. 2013). Given that optimal settlement environments differ substantially from light environments experienced by adults, potentially by as much as 10-fold (Suzuki et al. 2013), it is possible that variation in *Symbiodinium* communities between larvae and adults observed here relates to different selective pressures associated with differing light environments (Rowan et al. 1997; Gómez-Cabrera et al. 2008; Kemp et al. 2008). Other potentially numerous, uncharacterized differences between larval and adult microhabitats may also contribute to differences in selective pressures between life stages. The potential ecological roles for the larval-specific OTUs recorded here are unknown. Indeed, it is possible that they represent non-symbiotic, free-living types (Lee et al. 2016) that may have attached to the exterior of the larvae following release or that may have entered brooded larvae without engaging in symbiosis. Further work is needed to determine how many of these OTUs represent physiologically important versus transient *Symbiodinium*.

### Potential mechanisms shaping larval *Symbiodinium* communities

The immune system is an obvious mechanism by which the host could exert control over its symbiotic community by regulating the establishment of individual *Symbiodinium* types (Bay et al. 2011) or of either whole clades or functional units (i.e., clades or types with similar metabolic roles) (Ley et al. 2006). The symbioses of *Wolbachia* and *Spiroplasma* bacteria among *Drosophila* and lepidopteran genera, for example, are highly specific and exclude other bacterial lineages through a dynamic and mature immune response, to the extent that specific *Drosophila* species host novel and specific *Wolbachia* and/or *Spiroplasma* strains (Mateos et al. 2006; Russell et al. 2012). Mechanisms of immunity that could be transmitted through inheritance of parental genes include components of both the innate and adaptive immune response, including some that have been implicated in shaping invertebrate symbiont communities, such as T-cells, Nod2, defensins, and antimicrobial peptides (as reviewed in Franzenburg et al. 2013; Raina et al. 2016). These mechanisms have been documented during *Symbiodinium* establishment in corals (Wood-Charlson et al. 2006; Bay et al. 2011; Davy et al. 2012) and observed in the *Hydra*/bacteria symbiosis (Fraune et al. 2010; Franzenburg et al. 2013).

Conversely, the greater variation and diversity found in larval compared to adult *Symbiodinium* communities may be a function of an immature immune response that is not yet able to differentiate appropriate *Symbiodinium* types, rather than an adaptive response. As the coral immune system matures over time (Frank et al. 1997; Puill-Stephan et al. 2012), it is possible that a winnowing process eliminates symbionts that are not physiologically beneficial to the coral host (Abrego et al. 2009b; Byler et al. 2013). If true, then the ubiquitous presence of *Symbiodinium* C1_OTU136 in larvae may be a consequence of an opportunistic *Symbiodinium* type taking advantage of immature host immunity. Further work is needed to identify the role that the immune response has in shaping *Symbiodinium* communities; in particular what (if any) immune-related genes are being transmitted from parents to offspring and whether novel symbionts are a function of an under-developed immune response.

### Winnowing and microhabitat variation may shape adult *Symbiodinium* communities

The disparate *Symbiodinium* communities in larvae versus adults found here further indicate that the re-shaping of the *Symbiodinium* community through ontogeny is an important developmental process in corals. Ontogenetic variability in microbial communities (both *Symbiodinium* and bacteria) is common in both vertically and horizontally transmitting cnidarian species (Coffroth et al. 2001; Nyholm and McFall-Ngai 2004; Abrego et al. 2009a, 2009b; Apprill et al. 2009; Littman et al. 2009; Sharp et al. 2012; Padilla-Gamiño et al. 2012; Poland et al. 2013; Byler et al. 2013; Lema et al. 2014; Poland and Coffroth 2017; Quigley et al. 2017b). The low level of variation in *Symbiodinium* communities associated with corals ranging in diameter from 8 cm to >30 cm (3–10 years Babcock 1991) suggests that the end of the winnowing process likely occurs earlier in the development of the brooding coral *S. hystrix* (ShA) (i.e., before 3 years) than in broadcast-spawning corals (~3.5 years; Abrego et al. 2009a, 2009b). Although evidence for switching of symbiont communities in adults corals exists (Boulotte et al. 2016), the pre-winnowing period may be the most flexible time for hosts to associate with a diversity of microbes. Microhabitat variation in the abundances of C120, D1, and D1a in adult corals at meter-level scales found in this study could be important for structuring *in hospite Symbiodinium* diversity, and may be partly responsible for the variability found at the level of individual larvae and broods. Therefore, identifying at what stage winnowing occurs in brooding corals and the influence of fine-scale environmental variables will provide crucial insights into when the flexibility to associate with environmentally acquired and potentially stress-tolerant types diminishes and specialization of the *Symbiodinium* community begins.

## Conclusion

On the basis of novel heritability and paternity analyses, we show that *Symbiodinium* communities associated with the brooding coral *S. hystrix* (ShA) are only partially genetically regulated by their host and that larvae retain the flexibility to associate with novel symbionts across generations. Our results reveal a mixed-mode transmission strategy for establishing *Symbiodinium* communities in larvae of a brooding coral, based on demonstrations that a subset of novel and unique *Symbiodinium* types are found in brooded larvae but not in adults (although adults also had their own unique types). Importantly, this information aligns symbiosis transmission ecology in corals with well-known terrestrial invertebrate symbioses that typically exhibit mixed-mode transmission strategies. Advances in the understanding of heritable genetic mechanisms quantified here provide important insights into how *Symbiodinium* communities may be targeted for intervention strategies to increase reef resilience.

### Data accessibility

DNA sequences: All sequencing data will be made available through NCBI SRA SRP082130.

## Electronic supplementary material

Supporting Information
