# Supplementary material for: Unexpected mixed-mode transmission and moderate genetic regulation of Symbiodinium communities in a brooding coral
Source: Heredity (Edinb). 2018 Feb 17;121(6):524–36. doi: 10.1038/s41437-018-0059-0 (PMC6221883; doi:10.1038/s41437-018-0059-0)
Supplement: Supplementary file 1 — Supporting Information [file 41437_2018_59_MOESM1_ESM.docx]

**Supporting information**

**Material and Methods**

**Study species and sampling design**

Eighteen mature colonies were collected from the site one day before the predicted larval release period in November 2009. Brooding colonies were maintained in separate aquaria supplied with 1 µm filtered seawater at Lizard Island Research Station. Larvae were collected from the 18 colonies early in the morning, within 5 hours of release, every day for two weeks and preserved in 100% ethanol. Multilocus genotypes were generated from microsatellite markers for all adult colonies (including brooding dams) and larvae and used to assign paternity. Paternity assignments were then used to construct consensus confidence categories of paternity (Very High, High, Medium, Low) for each of the larvae genotyped in the current study. A full description of the site, extraction methods and parentage analysis can be found in (Warner *et al.*, 2016). Library preparation and sequencing were performed at the University of Texas at Austin’s Genomics Sequencing and Analysis Facility (USA). Quantity and quality of *Symbiodinium* DNA were determined using a Bioanalyzer (Agilent) to standardize the input of each sample to the same concentration for input into triplicate PCRs, with cycle checks after 23, 26, and 29 cycles (see Quigley et al. 2014 and Green et al. 2014 for specific PCR conditions, clean-up protocols, and further details of library preparation).

***Symbiodinium* community genotyping**

Chimeric reads were filtered and those with an Expected Error greater than 1.0 were discarded (Edgar and Flyvbjerg, 2015). To generate OTUs, remaining reads were clustered with the default 97% identity and minimum cluster size of 2, thus eliminating all singleton reads. Mapping was performed by globally aligning sequences with 99% similarity, with gaps counted as differences. This resulted in 927 OTUs, consisting of 4,689,233 reads (67.5% of original data) across the 105 samples (mean per sample = 44,659±1,940). OTUs were annotated through locally run BLAST+ searches against the complete non-redundant NCBI nt database. *Symbiodinium* specific reads made up a majority of the dataset with 4,638,791 (98.9% of the total cleaned reads) across 161 OTUs. OTUs that did not blast to *Symbiodinium* or host name accessions with an Expect value (E) greater than 0.001 (De Wit et al. 2012) were filtered and removed, totalling 50,442 reads (1.07% of the total cleaned reads) and 766 OTUs. Of the 161 OTUs (Table S2), 0.03% were identified in NCBI as sequences derived from mixed *S. hystrix*/*Symbiodinium* libraries (i.e. Blast accession information only states that DNA extracts were derived from host and symbiont tissue). Blast searches of unknown *Symbiodinium* types resulted in matches to studies that used either RFLP or qPCR to identity *Symbiodinium* samples to the clade level (i.e. clade C) and where therefore annotated as putative C types (Mayfield *et al.*, 2011, 2012, 2014). In general, blast hits were used to classify each OTU, first by clade and then, where possible, by type. To further classify to the type level, OTUs were re-blasted using Blastn with *Symbiodinium*-specific taxonomy search criteria (taxon id: 2949). Furthermore, clade-level OTUs were also aligned after manual editing with BioEdit (Hall, 1999) to known reference sequences available from the Santos Lab (<http://www.auburn.edu/~santosr/sequencedatasets.htm>) and from previous *Symbiodinium* phylogenetic analyses (LaJeunesse *et al.*, 2010). Additional reference sequences were added to these resources to represent the known *Symbiodinium* diversity for the *S. hystrix* species complex (C120: HM185737, C120a: HM185738, C1aa: HM185739, C3ff: HM185740, C1mHM185741, C3nt: EF541149) (Tonk *et al.*, 2013).

*Symbiodinium* OTUs from clades A-G were aligned separately (Baker and Correa 2009) using Clustal Omega (Sievers *et al.*, 2011). Trees were constructed in ClustalW2 Phylogeny with default settings (Larkin *et al.*, 2007; Goujon *et al.*, 2010; McWilliam *et al.*, 2013)(S1 Fig). All mean values are accompanied by their standard errors unless otherwise stated. *Symbiodinium* designations therefore follow the type designation followed by OTU number (e.g. C15_OTU46). Type designation is based on NCBI database searches and re-blasting to taxon id 2949, in which the taxon with the best blast hit above an E value greater than 0.001 was selected. If still uncharacterized, the OTU was aligned to standardized sequences.

**Multiple ITS-2 copies and intragenomic variation**

Comparisons between single-cell and next-generation sequencing suggests that clustering across samples at 97% similarity sufficiently collapses intragenomic variants to the type level (Arif *et al.*, 2014), as has been used in this study. Furthermore, a recent study suggests that clustering across samples at 97% identity underestimates diversity instead of overestimating it (Cunning *et al.*, 2017). Although it is not definitively clear whether clustering across samples within a single species underestimates diversity (given these conclusions were drawn by comparing *Symbiodinium* community diversity across six coral species), replicates at the species level for 4 of the 6 species in this study suggests that diversity is underestimated. Intragenomic variation and generation of false-positives is therefore substantially minimized by using across-sample clustering at 97% similarity, as we have employed. Single cell sequencing is currently financially and logistically outside the scope of studies that examine communities of hundreds of different *Symbiodinium* types (as with coral juveniles), with a majority of these types not yet existing in culture. It is questionable if microsatellite flanking regions provide superior taxonomic resolution (Howells *et al.*, 2016), and as no known single-copy marker exists, using other markers in tandem with ITS-2 will only result in data representing multiple, multi-copy markers. Overall, we undertook a three-step approach, as outlined in (Quigley *et al.*, 2016), to assess if multiple copies and intragenomic variation of ITS-2 genes could potentially bias abundance and heritability estimates across *Symbiodinium* types after clustering at 97% identity. Briefly, OTUs were first divided by clade and compared for co-occurrence across samples using the plot_tree function in ‘Phyloseq’ and grouped into subsets of co-occurring OTUs. Secondly, OTUs that increased proportionally (i.e., both OTUs increasingly in similar increments to each other) and with high percent pairwise similarity relative to each other were compared. Finally, pairwise percent identities were calculated for these latter subsets of OTUs using the package ‘Ape’ (Paradis *et al.*, 2004), and correlations of variance-normalized abundances were calculated for pairs that had greater than 85% similarity with the function ggpairs in the package ‘GGally’ (Schloerke *et al.*, 2014). Only two groups of OTUs fulfilled these three criteria, suggested that these are the most likely intragenomic candidates (S1 Fig). However, to be conservative, the diversity metric was calculated taking into account all possible intragenomic variation by pooling the raw abundances of potential intragenomic variants (OTUs: 8/10, 12/22/24, 28/223, 3/6, 588/848), and heritability was calculated using the parameters described above.

**Results**

***Symbiodinium* community changes across maternal larval broods**

The occurrence and abundance of OTUs in larvae that shared colony 2 as a dam did not differ significantly from those of larvae in broods from dams 3, 4 or 18. In contrast, broods from these 4 dams (2, 3, 4, 18) were associated with seven different OTUs compared to broods from dams 6, 7, 13, and 14. Larvae from dam 2 had between 1.5-2.4-log2 fold greater abundance of A3 and A1 compared to broods of dams 6, 7, 13 and 14 (Table S5, *p-adjusted*: 0.04 - 4 e^-05^). They also had almost 2-log2 fold greater abundances of D1 and D1a (OTUs 3 and 6) than dam 13 broods, 5.4-log2 fold less C-type OTU55 and 7-log2 fold more C1-OTU2 (*p-adjusted*: 9.6 e^-03^- 4.8 e^-03^). The dam 2 brood also had 2.7-log2 fold less C1-OTU32 (*p-adjusted*: 0.03). The composition of *Symbiodinium* communities associated with larvae from dam 3’s brood was comparable to that of many other families, but did differ significantly from *Symbiodinium* communities associated with dam 4’s larvae, by having 3.5-5.9-log2 fold less of two C-types (OTU2 and 44), and from dam13’s larvae, by having 4.4-log2 fold less C1-OTU2 (*p-adjusted:* 0.0001 - 0.02). C1v1e-OTU44 distinguished dam 4’s larvae from those of other dams, occurring in 5.2-7-log2 fold greater abundances in dam 4 larvae compared to broods from dams 6, 7,10, 14, and 18 (*p-adjusted:* 2.46 e^-07^ - 6.08 e^-03^). There was also 1.1-1.4-log2 fold lower abundance of C31- OTU733 in larvae from dam 4 compared to larvae from dams 6 and 14, and 2-2.6 lower abundance of clade C1- OTU7 than in larvae from dams 6 and 13 (*p-adjusted:* 6.59 e^-03^ - 3.04 e^-02^). Larvae of dam 4 did have a log2 fold more C1-OTU134 than larvae of dam 6 (*p-adjusted:* 1.13 e^-02^). Finally, dam 13 was the most different in abundance compared to dam 4 due to varying abundances of clade C OTUs 2, 4, 21, and log2 fold increases in D1a-OTU6, D1-OTU3 and A3-OTU8 (Table S5). In conjunction with the differences discussed above, the dam 6 brood also had differential abundances compared to dam broods 7, 13, and 18 across seven clade C *Symbiodinium*, with each brood having log2 fold differences corresponding to unique C-types. The only exception was C1-OTU4, in which brood 6 had significantly less of this clade C type compared to either 13 or 18 (*p-adjusted:* 3.2 e^-03^ - 0.05). There were no differences compared to dam broods 10 or 14. Dam 7 broods had over 7-log2 fold greater abundances of clade C1-OTU2 (*p-adjusted:* 3.2 e^-42^). Larvae produced from dam 10 were distinct from brood 13 with 1.4-5.9-log2 fold greater abundances two C-types (OTU1, OTU2) as well as previously mentioned differences with other broods (*p-adjusted:* 5.3 e^-08^ - 4.9 e^-02^). Dam 13 larvae had large log2 fold differences in abundance of C types OTU2 and 4 compared to dam 14 and brood 18 due to the abundance of three C, two D and one A type. Finally, dam 14 larvae significantly differed in abundance compared to broods 2, 4, 13, and 18 in their 5-log2 fold lower abundance of clade C1-OTU4 (*p-adjusted:* 0.008).

**Heritability**

The mean and mode of posterior heritability distribution increased by incorporating intragenomic variants (mean: 0.45 ± 0.21 SD; mode 0.38 BCI: 0.1-0.9). Even with a moderate sample size (n=60), the majority of heritability estimates in the posterior distribution of the heritability estimate had a greater mode than the one estimated, suggesting that the most probable values of h^2^ are greater than the estimate derived here (Fig 2).

**Multiple ITS-2 copies and intragenomic variation**

**Analysis of all *Symbiodinium* OTUs:** The level of co-occurrence of OTUs, correlations of proportional abundance and percent pairwise similarity did not reveal evidence of ITS-2 intragenomic multicopy signatures in any of the *Symbiodinium* clades retrieved from *S. hystrix* (ShA). Of the seven clade A sequences, only A1_OTU10 and A3_OTU8 co-occurred across samples and had strongly correlated relative proportions (R^2^ = 0.853). However, both were identified as distinct A types (e-values: 2.00E-155 and 1.00E-157) with a percent pairwise similarity of 49.8%. Twenty-six of the 62 C-type OTUs co-occurred and 39 of 676 comparisons had proportional correlations greater than R^2^ = 0.5. However, only three comparisons had R^2^ values > 0.80 (OTU22 vs. OTU12: 0.82, OTU12 vs. OTU24: 0.83, OTU223 vs. OTU28: 0.85) and none of these pairs had sequence similarity greater than 51.3% nucleotide identity. Three of the 7 clade D OTUs co-occurred and the abundance of D1 was moderately correlated with the abundance of D1a (R^2^=0.761) but these OTUs were only 58.8% similar. Only one comparison between the four clade E OTUs (588 vs 848) resulted in a large proportional correlation (R^2^ = 0.71) but OTU848 was only found in one of the four samples that contained OTU588 and they shared 37.1% nucleotide identity. The two clade G OTUs were not found in any of the same samples (R^2^=-0.24).

To evaluate the likelihood that the OTUs that differed significantly in their abundance between larvae and adults, among maternal broods and colony size classes (35, 15 and 4 OTUs) were multiple copies of the ITS-2 gene, we analysed their co-occurrence and proportional abundance, as described above. A1*_*OTU10*,* A3_OTU8, D1_OTU3, and D1a_OTU6 were significantly different between larvae and adults and among broods, and are unlikely to be intra-genomic variants. D1_OTU597 was found to only differ between larvae and adults. D1_OTU3 and D1a_OTU6 always co-occurred (but not always proportionally), thus making them reasonable intragenomic variants candidates. Recent work combining multiple markers and microsatellites has distinguished D1 as *S. glynni* and D1a as *S. trenchi,* despite vestiges of D1 ITS-2 being found within the genome of D1a (LaJeunesse *et al.*, 2010, 2014). Finally, D1_OTU597 was also found in 56 of 106 samples in much lower abundances than the other two D OTUs. If D1_OTU597 is an intragenomic variant of either *S. glynnii* or *S.trenchii,* then it should increase proportionally with an increase in either of these OTUs. This was not the case (R^2^ correlation coefficients D1_OTU3: 0.5, D1a_OTU6: 0.3). From clade C, there were 24 and 9 OTUs that differed significantly between larvae and adults and amongst maternal broods, totalling unique 25 OTUs (8 OTUs were found in both categories). The C OTUs 12, 22 and 24 showed signs of strong proportional increase/decrease (R^2^= 0.82-0.85), but only OTUs 223 and 28 had a high correlation coefficients (R^2^=0.85). The abundances of all OTUs described above differed between larvae and adults.

**References**

Wit P, Pespeni MH, Ladner JT, Barshis DJ, Seneca F, Jaris H, Therkildsen NO, Morikawa M, Palumbi SR (2012). The simple fool's guide to population genomics via RNA‐Seq: an introduction to high‐throughput sequencing data analysis. *Mol Eco Res* 1;12(6):1058-67.

Edgar RC, Flyvbjerg H (2015). Error filtering, pair assembly and error correction for next-generation sequencing reads. *Bioinformatics*: btv401.

Goujon M, McWilliam H, Li W, Valentin F, Squizzato S, Paern J, *et al.* (2010). A new bioinformatics analysis tools framework at EMBL–EBI. *Nucleic Acids Res* **38**: W695–W699.

Green, E.A., Davies, S.W., Matz, M.V. and Medina, M. (2014). Quantifying cryptic *Symbiodinium* diversity within *Orbicella faveolata* and *Orbicella franksi* at the Flower Garden Banks, Gulf of Mexico. *PeerJ*, 2, p.e386.

Hall TA (1999). BioEdit: a user-friendly biological sequence alignment editor and analysis program for Windows 95/98/NT. In: *Nucleic acids symposium series*, Vol 41, pp 95–98.

LaJeunesse TC, Pettay DT, Sampayo EM, Phongsuwan N, Brown B, Obura DO, *et al.* (2010). Long-standing environmental conditions, geographic isolation and host–symbiont specificity influence the relative ecological dominance and genetic diversification of coral endosymbionts in the genus *Symbiodinium*. *J Biogeogr* **37**: 785–800.

LaJeunesse TC, Wham DC, Pettay DT, Parkinson JE, Keshavmurthy S, Chen CA (2014). Ecologically differentiated stress-tolerant endosymbionts in the dinoflagellate genus *Symbiodinium* (Dinophyceae) Clade D are different species. *Phycologia* **53**: 305–319.

Larkin MA, Blackshields G, Brown NP, Chenna R, McGettigan PA, McWilliam H, *et al.* (2007). Clustal W and Clustal X version 2.0. *Bioinformatics* **23**: 2947–2948.

Mayfield AB, Chan P-H, Putnam HM, Chen C-S, Fan T-Y (2012). The effects of a variable temperature regime on the physiology of the reef-building coral Seriatopora hystrix: results from a laboratory-based reciprocal transplant. *J Exp Biol* **215**: 4183–4195.

Mayfield AB, Chen YH, Dai CF, Chen CS (2014). The effects of temperature on gene expression in the Indo-Pacific reef-building coral Seriatopora hystrix: insight from aquarium studies in Southern Taiwan. *Int J Mar Sci* **4**.

Mayfield AB, Wang L-H, Tang P-C, Fan T-Y, Hsiao Y-Y, Tsai C-L, *et al.* (2011). Assessing the impacts of experimentally elevated temperature on the biological composition and molecular chaperone gene expression of a reef coral. *PLoS One* **6**: e26529.

McWilliam H, Li W, Uludag M (2013). Analysis Tool Web Services from the EMBL-EBI Nucleic acids research: 41 (Web Server issue): W597-600. 16.

Quigley KM, Davies SW, Kenkel CD, Willis BL, Matz M V, Bay LK (2014). Deep-sequencing method for quantifying background abundances of *Symbiodinium* types: exploring the rare *Symbiodinium* biosphere in reef-building corals. *PLoS One* **9**: e94297.

Quigley KM, Willis BL, Bay LK (2016). Maternal effects and *Symbiodinium* community composition drive differential patterns in juvenile survival in the coral *Acropora tenuis*. *R Soc Open Sci* **3**: 1–17.

Sievers F, Wilm A, Dineen D, Gibson TJ, Karplus K, Li W, *et al.* (2011). Fast, scalable generation of high‐quality protein multiple sequence alignments using Clustal Omega. *Mol Syst Biol* **7**.

Tonk L, Bongaerts P, Sampayo EM, Hoegh-Guldberg O (2013). SymbioGBR: a web-based database of *Symbiodinium* associated with cnidarian hosts on the Great Barrier Reef. *BMC Ecol* **13**: 7.

Warner PA, Willis BL, van Oppen MJH (2016). Sperm dispersal distances estimated by parentage analysis in a brooding scleractinian coral. *Mol Ecol* **25**: 1398–1415.


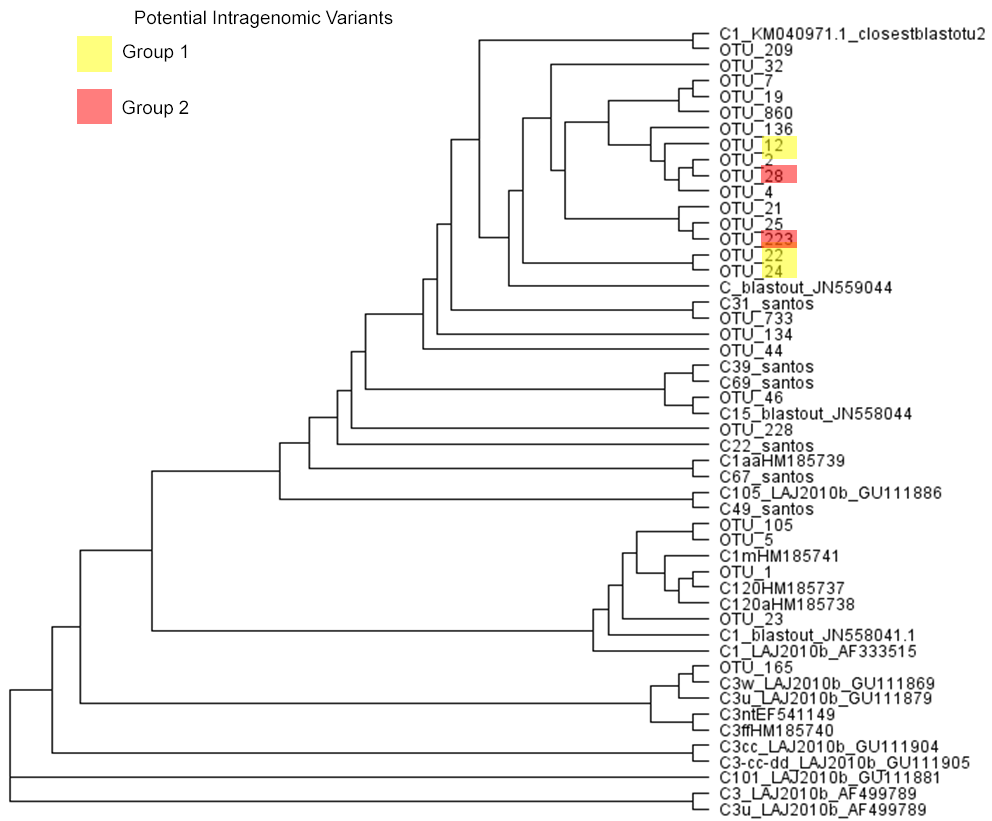


**Figure S1.** ClustalW2 Neighbour-joining cladogram showing all OTUs with significant *p-adjusted* values for the following groups: planula and adults, maternal broods, and size classes. Additional sequences are references of *Symbiodinium* types known to inhabit ShA or those whose genetic diversification has been investigated (tip labels without “OTU”).


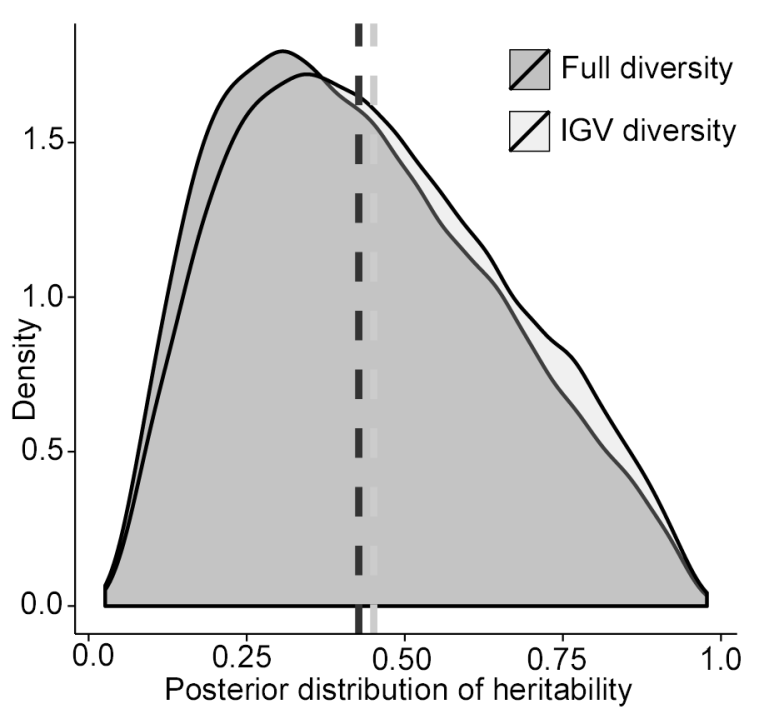


**Figure S2.** The posterior Bayesian heritability distributions using full diversity metric (dark grey) and intragenomic variants (IGV, light grey). The dashed lines represent the mean narrow-sense heritability, with the colour corresponding to the type of diversity metric used.

**Table S1.** Pedigree information for each of the 60 ShA larvae. Each dam and sire number represents a unique colony. ShA are hermaphroditic and the nine dams were therefore also represented as sires.

| **Larval Id** | **Dam** | **Sire** |
| --- | --- | --- |
| 2.1.6 | 2 | 115 |
| 2.1.9 | 2 | 45 |
| 2.2.11 | 2 | 79 |
| 2.2.12 | 2 | 31 |
| 2.2.2 | 2 | 111 |
| 2.2.3 | 2 | 3 |
| 2.2.6 | 2 | 16 |
| 3.1.26 | 3 | 97 |
| 4.1.6 | 4 | 81 |
| 4.2.11 | 4 | 5 |
| 4.2.8 | 4 | 9 |
| 4.2.9 | 4 | 16 |
| 4.4.14 | 4 | 26 |
| 4.4.1 | 4 | 5 |
| 4.4.2.17 | 4 | 5 |
| 4.4.3 | 4 | 103 |
| 6.1.1 | 6 | 33 |
| 6.2.12 | 6 | 64 |
| 6.2.18 | 6 | 6 |
| 6.2.20 | 6 | 10 |
| 6.2.5 | 6 | 154 |
| 6.4.12 | 6 | 18 |
| 6.4.17 | 6 | 73 |
| 6.4.1 | 6 | 60 |
| 6.5.2 | 6 | 79 |
| 6.5.4 | 6 | 160 |
| 6.6.11 | 6 | 4 |
| 6.6.19 | 6 | 64 |
| 6.6.1 | 6 | 66 |
| 6.6.20 | 6 | 97 |
| 7.1.1 | 7 | 2 |
| 7.3.18 | 7 | 2 |
| 7.3.19 | 7 | 32 |
| 7.3.24 | 7 | 79 |
| 7.3.25 | 7 | 33 |
| 7.3.3 | 7 | 3 |
| 7.3.5 | 7 | 116 |
| 7.3.7 | 7 | 2 |
| 7.3.8 | 7 | 7 |
| 10.2.12 | 10 | 113 |
| 13.2.12 | 13 | 125 |
| 13.2.3 | 13 | 60 |
| 13.2.9 | 13 | 113 |
| 13.3.10 | 13 | 167 |
| 13.3.3 | 13 | 170 |
| 13.3.4 | 13 | 109 |
| 13.3.9 | 13 | 39 |
| 14.1.10 | 14 | 14 |
| 14.1.11 | 14 | 14 |
| 14.1.13 | 14 | 14 |
| 14.1.18 | 14 | 113 |
| 14.1.23 | 14 | 8 |
| 18.1.4 | 18 | 96 |
| 18.2.12 | 18 | 13 |
| 18.2.3 | 18 | 153 |
| 18.2.4 | 18 | 121 |
| 18.2.6 | 18 | 99 |
| 18.3.10 | 18 | 63 |
| 18.3.11 | 18 | 157 |
| 18.3.5 | 18 | 17 |

**Table S2.** Final abundance of cleaned reads assigned to each *Symbiodinium* clade and their percent abundance and OTU number across all clades.

| **Clade** | **Reads** | **Proportion of cleaned (%)** | **OTUs** |
| --- | --- | --- | --- |
| A | 69775 | 1.5 | 7 |
| B | 47 | 0.001 | 1 |
| C | 4,393,359 | 94.7 | 62 |
| D | 173,549 | 3.7 | 7 |
| E | 106 | 0.002 | 4 |
| G | 496 | 0.01 | 2 |
| Putative C | 1459 | 0.03 | 78 |
| Total | 4,638,791 | - | 161 |

**Table S3.** A large number of clade C identified OTUs could not be identified to the type level using a general Blast search against the whole NCBI nt database. In order to increase taxonomic resolution, we re-blasted OTUs to a *Symbiodinium* specific database (taxon id: 2949) as well as aligned using ClustalW them to known standards for this species (Tonk *et al.*, 2013). We have summarized the taxonomic designations here for only those OTUs that we found to be significantly differently abundant (*p-adjusted)* between our comparisons of interest (adult vs. planula; between maternal broods; adult size classes).

| **OTU name:** | **Blast identity:** | **Blast E-value for general/ *Symbiodinium* specific search:** | **Accession N°:** | **Phylogenetic designation:** |
| --- | --- | --- | --- | --- |
| OTU1 | C1 | 2.00e-168 | JN558041 | C120, C120a |
| OTU2 | C1 | 2e-33 | KM041000.1/KM040971.1 | C |
| OTU4 | C1 | 5e-35 | KM041000.1/KM040971.1 | C |
| OTU5 | C1 | 3.00e-166 | JN558041 | C1m |
| OTU7 | C1 | 3e-24 | KM041000.1 | C |
| OTU12 | C1 | 2e-34 | KM041000.1 | C |
| OTU19 | C1 | 1e-22 | KM041000.1 | C |
| OTU21 | C1 | 2e-39 | KM041000.1 | C |
| OTU22 | C1 | 1e-30 | KM041000.1 | C |
| OTU23 | C1 | 1e-156 | JN558041.1 | C1 |
| OTU24 | C1 | 2e-26 | KM041000.1 | C |
| OTU25 | C1 | 1e-30 | KM041000.1 | C |
| OTU28 | C1 | 4e-29 | KM041000.1 | C |
| OTU32 | C1 | 2e-26 | KM041000.1 | C |
| OTU44 | C1v1e | 1e-149 | HG942431.1 | - |
| OTU46 | C15 | 2.00e-168 | JN558044 | C15 |
| OTU105 | C1 | 1.00e-151 | JN558041 | C1m |
| OTU134 | C1 | 6.00e-162 | JN558041 | - |
| OTU136 | C1 | 2e-33 | KM041000.1 | C |
| OTU165 | C1 | 1.00E-163 | KM041000.1 | C3w |
| OTU209 | C | 3e-06 | AB294631.1 | C1 |
| OTU223 | C1 | 4e-29 | KM041000.1 | C |
| OTU228 | C1v6 | 5e-175 | HG942433.1 | C22 |
| OTU733 | C1 | 4.00e-113 | JN558041 | C31 |
| OTU860 | C1 | 2e-21 | KM041000.1 | C |

**Table S4.** Summary table for differential abundance testing using DESeq2 comparing *Symbiodinium* communities in adults and planula. Values were derived from negative binomial models. Padj value represents Bonferroni adjusted p-values.

| **Comparison** | **OTU** | **Identity** | **baseMean** | **log2FoldChange** | **Padj** |
| --- | --- | --- | --- | --- | --- |
| Adult v planula | OTU22 | C/C1 | 26.1 | -5.1 | 1.17 e -48 |
| Adult v planula | OTU3 | D1 | 848.8 | 2.1 | 5.67 e -30 |
| Adult v planula | OTU2 | C/C1 | 2671.1 | -3.8 | 4.72 e -28 |
| Adult v planula | OTU10 | A1 | 137.6 | 2.2 | 4.1 e -24 |
| Adult v planula | OTU8 | A3 | 231.2 | 2 | 4.3 e -24 |
| Adult v planula | OTU12 | C/C1 | 79.2 | -2.9 | 3.9 e -19 |
| Adult v planula | OTU24 | C/C1 | 31.7 | -3.1 | 1.67 e -18 |
| Adult v planula | OTU7 | C/C1 | 122.4 | -3.9 | 6.67 e -17 |
| Adult v planula | OTU165 | C1/C3w | 68.3 | 1 | 6.67 e -17 |
| Adult v planula | OTU32 | C/C1 | 3.6 | -5.4 | 1.74 e -16 |
| Adult v planula | OTU6 | D1a | 75.6 | 2.2 | 3.3 e -16 |
| Adult v planula | OTU25 | C/C1 | 1.3 | -3.4 | 1.5 e -11 |
| Adult v planula | OTU28 | C/C1 | 18.2 | -3.4 | 1.07 e -10 |
| Adult v planula | OTU597 | D1 | 0.93 | 2.5 | 1.07 e -09 |
| Adult v planula | OTU23 | C1 | 19.8 | 0.87 | 5.95 e -09 |
| Adult v planula | OTU105 | C1/C1m | 113.1 | 0.74 | 2.1 e -08 |
| Adult v planula | OTU4 | C/C1 | 846.2 | -3.4 | 2.54 e -08 |
| Adult v planula | OTU223 | C/C1 | 10 | -3 | 4.01 e -07 |
| Adult v planula | OTU1 | C1/C120, C120a | 23,094.40 | 0.48 | 2.97 e -06 |
| Adult v planula | OTU21 | C/C1 | 12.3 | -4.8 | 4.6 e -06 |
| Adult v planula | OTU5 | C1/C1m | 169 | 0.47 | 5.5 e -05 |
| Adult v planula | OTU228 | C/C1v6/C22 | 18 | 0.77 | 8.2 e -05 |
| Adult v planula | OTU19 | C/C1 | 17.2 | -3.1 | 2.42 e -03 |
| Adult v planula | OTU733 | C31/C1 | 67.4 | 0.54 | 5.49 e -03 |
| Adult v planula | OTU209 | C/C1 | 0.16 | -3.2 | 1.73 e -02 |
| Adult v planula | OTU860 | C/C1 | 0.16 | -3.2 | 2.16 e -02 |
| Adult v planula | OTU46 | C15 | 0.59 | 3.2 | 3.26 e -02 |
| Adult v planula | OTU134 | C1 | 72.9 | 0.44 | 3.43 e -02 |

**Table S5.** Summary table for differential abundance testing using DESeq2 comparing *Symbiodinium* communities in larval broods differing by dam. Values were derived from negative binomial models. Padj value represents Bonferroni adjusted p-values.

| **Comparison** | **OTU** | **Identity** | **baseMean** | **log2FoldChange** | **Padj** |
| --- | --- | --- | --- | --- | --- |
| Lv2 vs Lv6 | OTU10 | A1 | 52.2 | 1.7 | 0.002 |
| Lv2 vs Lv6 | OTU8 | A3 | 98.1 | 1.5 | 0.01 |
| Lv2 vs Lv6 | OTU32 | C/C1 | 5.9 | -2.8 | 0.03 |
| Lv2 vs Lv7 | OTU10 | A1 | 52.2 | 1.9 | 0.003 |
| Lv2 vs Lv13 | OTU2 | C/C1 | 4396.4 | 7 | 5.05 e -35 |
| Lv2 vs Lv13 | OTU8 | A3 | 98.1 | 2.4 | 4.00 e -05 |
| Lv2 vs Lv13 | OTU10 | A1 | 52.2 | 2.2 | 2.33 e -04 |
| Lv2 vs Lv13 | OTU44 | C/C1v1e | 2.2 | -5.5 | 9.61 e -04 |
| Lv2 vs Lv13 | OTU3 | D1 | 346.5 | 1.7 | 4.85 e -03 |
| Lv2 vs Lv13 | OTU6 | D1a | 30 | 2 | 9.63 e -03 |
| Lv2 vs Lv14 | OTU10 | A1 | 52.2 | 2.1 | 0.01 |
| Lv2 vs Lv14 | OTU8 | A3 | 98.1 | 1.8 | 0.04 |
| Lv3 vs Lv4 | OTU44 | C/C1v1e | 2.2 | -5 | 0.006 |
| Lv3 vs Lv4 | OTU2 | C/C1 | 4396.4 | -3.5 | 0.02 |
| Lv3 vs Lv13 | OTU2 | C/C1 | 4396.4 | 4.4 | 0.00019 |
| Lv4 vs Lv6 | OTU44 | C/C1v1e | 2.2 | 7 | 2.46 e -07 |
| Lv4 vs Lv6 | OTU134 | C1 | 61.7 | 1 | 1.13 e -02 |
| Lv4 vs Lv6 | OTU733 | C31/C1 | 54.4 | -1.1 | 1.76 e -02 |
| Lv4 vs Lv6 | OTU7 | C/C1 | 197.1 | -2 | 1.90 e -02 |
| Lv4 vs Lv7 | OTU44 | C/C1v1e | 2.2 | 5.4 | 0.0003 |
| Lv4 vs Lv10 | OTU44 | C/C1v1e | 2.2 | 5.5 | 0.002 |
| Lv4 vs Lv13 | OTU2 | C/C1 | 4396.4 | 7.9 | 1.35 e -47 |
| Lv4 vs Lv13 | OTU7 | C/C1 | 197.1 | -2.6 | 6.59 e -03 |
| Lv4 vs Lv13 | OTU6 | D1a | 30 | 1.7 | 3.04 e -02 |
| Lv4 vs Lv13 | OTU4 | C/C1 | 1289.7 | -3.3 | 4.09 e -02 |
| Lv4 vs Lv13 | OTU3 | D1 | 346.5 | 1.3 | 4.16 e -02 |
| Lv4 vs Lv13 | OTU8 | A3 | 98.1 | 1.2 | 4.97 e -02 |
| Lv4 vs Lv13 | OTU21 | C/C1 | 21.6 | -3.7 | 4.97 e -02 |
| Lv4 vs Lv14 | OTU44 | C/C1v1e | 2.2 | 5.3 | 0.0060767 |
| Lv4 vs Lv14 | OTU733 | C31/C1 | 54.4 | -1.4 | 0.0314999 |
| Lv4 vs Lv18 | OTU44 | C/C1v1e | 2.2 | 6.8 | 3.73 e -05 |
| Lv3 vs Lv4 | OTU44 | C/C1v1e | 2.2 | -5 | 0.006 |
| Lv3 vs Lv4 | OTU2 | C/C1 | 4396.4 | -3.5 | 0.02 |
| Lv3 vs Lv13 | OTU2 | C/C1 | 4396.4 | 4.4 | 0.00019 |
| Lv4 vs Lv6 | OTU44 | C/C1v1e | 2.2 | 7 | 2.46 e -07 |
| Lv4 vs Lv6 | OTU134 | C1 | 61.7 | 1 | 1.13 e -02 |
| Lv4 vs Lv6 | OTU733 | C31/C1 | 54.4 | -1.1 | 1.76 e -02 |
| Lv4 vs Lv6 | OTU7 | C/C1 | 197.1 | -2 | 1.90 e -02 |
| Lv4 vs Lv7 | OTU44 | C/C1v1e | 2.2 | 5.4 | 3.09 e -04 |
| Lv4 vs Lv10 | OTU44 | C/C1v1e | 2.2 | 5.5 | 2.40 e -03 |
| Lv4 vs Lv13 | OTU2 | C/C1 | 4396.4 | 7.9 | 1.35 e -47 |
| Lv4 vs Lv13 | OTU7 | C/C1 | 197.1 | -2.6 | 6.59 e -03 |
| Lv4 vs Lv13 | OTU6 | D1a | 30 | 1.7 | 3.04 e -02 |
| Lv4 vs Lv13 | OTU4 | C/C1 | 1289.7 | -3.3 | 4.09 e -02 |
| Lv4 vs Lv13 | OTU3 | D1 | 346.5 | 1.3 | 4.16 e -02 |
| Lv4 vs Lv13 | OTU8 | A3 | 98.1 | 1.2 | 4.97 e -02 |
| Lv4 vs Lv13 | OTU21 | C/C1 | 21.6 | -3.7 | 4.97 e -02 |
| Lv4 vs Lv14 | OTU44 | C/C1v1e | 2.2 | 5.3 | 6.08 e -03 |
| Lv4 vs Lv14 | OTU733 | C31/C1 | 54.4 | -1.4 | 3.15 e -02 |
| Lv4 vs Lv18 | OTU44 | C/C1v1e | 2.2 | 6.8 | 3.73 e -05 |
| Lv6 vs Lv7 | OTU1 | C1/ C120, C120a | 19319.9 | -0.7 | 0.02 |
| Lv6 vs Lv7 | OTU105 | C1/C1m | 85.1 | 0.8 | 0.03 |
| Lv6 vs Lv13 | OTU2 | C/C1 | 4396.4 | 6.8 | 2.20 e -42 |
| Lv6 vs Lv13 | OTU44 | C/C1v1e | 2.2 | -5.3 | 1.67e -03 |
| Lv6 vs Lv13 | OTU4 | C/C1 | 1289.7 | -4.5 | 3.22 e -03 |
| Lv6 vs Lv18 | OTU4 | C/C1 | 1289.7 | -3.3 | 0.05 |
| Lv6 vs Lv18 | OTU32 | C/C1 | 5.95 | 2.9 | 0.05 |
| Lv6 vs Lv18 | OTU81 | Putative C | 0.96 | 4.5 | 0.05 |
| Lv6 vs Lv18 | OTU134 | C1 | 61.7 | -0.9 | 0.05 |
| Lv7 vs Lv13 | OTU2 | C/C1 | 4396.4 | 7.3 | 3.2 e -42 |
| Lv10 vs Lv13 | OTU2 | C/C1 | 4396.4 | 5.9 | 5.3 e -08 |
| Lv10 vs Lv13 | OTU1 | C1/ C120, C120a | 19319.9 | 1.4 | 4.9 e -02 |
| Lv13 vs Lv14 | OTU2 | C/C1 | 4396.4 | -6.7 | 7.15 e -28 |
| Lv13 vs Lv14 | OTU4 | C/C1 | 1289.7 | 6.1 | 2.21 e -05 |
| Lv13 vs Lv18 | OTU2 | C/C1 | 4396.4 | -7 | 5.96 e -38 |
| Lv13 vs Lv18 | OTU8 | A3 | 98.1 | -1.8 | 4.61 e -03 |
| Lv13 vs Lv18 | OTU44 | C/C1v1e | 2.2 | 5.1 | 4.61 e -03 |
| Lv13 vs Lv18 | OTU21 | C/C1 | 21.6 | 5.3 | 5.47 e -03 |
| Lv13 vs Lv18 | OTU6 | D1a | 30 | -1.9 | 8.59 e -03 |
| Lv13 vs Lv18 | OTU3 | D1 | 346.5 | -1.3 | 3.80 e -02 |
| Lv14 vs Lv18 | OTU4 | C/C1 | 1289.7 | -4.9 | 0.008 |

**Table S6.** Summary table for differential abundance testing using DESeq2 comparing *Symbiodinium* communities in adult colony size classes. Values were derived from negative binomial models. Padj value represents Bonferroni adjusted p-values.

| **Comparison** | **OTU** | **Identity** | **baseMean** | **log2FoldChange** | **Padj** |
| --- | --- | --- | --- | --- | --- |
| 8-14 vs 14-20 | OTU4 | C/C1 | - | 3.18 | 0.001 |

**Table S7.** Summary table of the full OTU names and their abbreviations depicted in Figure 1. For figure clarity, only 83 points are depicted on this NMDS. These include those OTUs with the highest confidence as belonging to the genus *Symbiodinium* (i.e., OTUs that had accessions with an Expect value (E) greater than 0.001 to a specific *Symbiodinium* identity. Those that were excluded from this figure were those that blasted with the same E-value threshold but were only identified to host/symbiont mixed identities (i.e. “*S. hystrix*/*Symbiodinium* libraries”). The following are shown: 24 of the 93 OTUs unique to larvae, 16 of the 17 OTUs unique to adult, and 43 of the 51 OTUs shared between larvae and adult.

| OTU name | Clade |
| --- | --- |
| OTU45_A | A |
| OTU196_A | A |
| OTU511_A13 | A |
| OTU8_A3 | A |
| OTU554_A3 | A |
| OTU10_micro | A |
| OTU129_micro | A |
| OTU181_B1 | B |
| OTU24_C | C |
| OUT27_C | C |
| OTU44_C | C |
| OTU91_C | C |
| OTU193_C | C |
| OTU209_C | C |
| OTU228_C | C |
| OTU252_C | C |
| OTU285_C | C |
| OTU517_C | C |
| OTU530_C | C |
| OTU569_C | C |
| OTU571_C | C |
| OTU724_C | C |
| OTU731_C | C |
| OTU740_C | C |
| OTU743_C | C |
| OTU750_C | C |
| OTU760_C | C |
| OTU22_C | C |
| OTU23_C1 | C |
| OTU29_C1 | C |
| OTU47_C1 | C |
| OTU51_C1 | C |
| OTU58_C1 | C |
| OTU105_C1 | C |
| OTU134_C1 | C |
| OTU165_C1 | C |
| OTU437_C1 | C |
| OTU464_C1 | C |
| OTU730_C1 | C |
| OTU733_C1 | C |
| OTU736_C1 | C |
| OTU741_C1 | C |
| OTU1_C1 | C |
| OTU5_C1 | C |
| OTU46_C15 | C |
| OTU451_C15 | C |
| OTU2_C_variant | C |
| OTU53_C_variant | C |
| OTU86_C_variant | C |
| OTU136_C_variant | C |
| OTU184_C_variant | C |
| OTU223_C_variant | C |
| OTU432_C_variant | C |
| OTU562_C_variant | C |
| OTU783_C_variant | C |
| OTU833_C_variant | C |
| OTU846_C_variant | C |
| OTU4_C_variant | C |
| OTU860_C_variant | C |
| OTU881_C_variant | C |
| OTU886_C_variant | C |
| OTU169_C_variant | C |
| OTU589_C_variant | C |
| OTU7_C_variant | C |
| OTU12_C_variant | C |
| OTU19_C_variant | C |
| OTU21_C_variant | C |
| OTU25_C_variant | C |
| OTU28_C_variant | C |
| OTU32_C_variant | C |
| OTU3_D1 | D |
| OTU120_D1 | D |
| OTU597_D1 | D |
| OTU904_D1 | D |
| OTU6_D1a | D |
| OTU201_D1a | D |
| OTU803_D1a | D |
| OTU75_E | E |
| OTU340_E_foram | E |
| OTU588_E | E |
| OTU848_E_foram | E |
| OTU34_G4 | G |
| OTU200_G6 | G |
